# Artificially designed pathogens – a diagnostic option for future military deployments

**DOI:** 10.1186/s40779-015-0045-2

**Published:** 2015-07-09

**Authors:** Andreas E. Zautner, Wycliffe O. Masanta, Rebecca Hinz, Ralf Matthias Hagen, Hagen Frickmann

**Affiliations:** Institut für Medizinische Mikrobiologie, Universitätsmedizin Göttingen, Göttingen, Germany; Fachbereich Tropenmedizin am Bernhard-Nocht Institut, Bundeswehrkrankenhaus Hamburg, Hamburg, Germany; Institut für Mikrobiologie, Virologie und Hygiene, Universitätsmedizin Rostock, Rostock, Germany

**Keywords:** Deployment, Sequencing, Next generation sequencing, DNA shuffling, Synthesis, Diagnosis, Synthetic biology

## Abstract

Diagnostic microbial isolates of bio-safety levels 3 and 4 are difficult to handle in medical field camps under military deployment settings. International transport of such isolates is challenging due to restrictions by the International Air Transport Association. An alternative option might be inactivation and sequencing of the pathogen at the deployment site with subsequent sequence-based revitalization in well-equipped laboratories in the home country for further scientific assessment.

A literature review was written based on a PubMed search.

First described for poliovirus in 2002, *de novo* synthesis of pathogens based on their sequence information has become a well-established procedure in science. Successful syntheses have been demonstrated for both viruses and prokaryotes. However, the technology is not yet available for routine diagnostic purposes.

Due to the potential utility of diagnostic sequencing and sequence-based *de novo* synthesis of pathogens, it seems worthwhile to establish the technology for diagnostic purposes over the intermediate term. This is particularly true for resource-restricted deployment settings, where safe handling of harmful pathogens cannot always be guaranteed.

## Introduction

During military deployment, close proximity of individuals within field camps, restricted hygiene options during more robust military operations, new environments and interactions between people and animals on site can lead to contagion with an infectious disease followed by an outbreak in the camp [1–8]. This pattern has been observed in a number of military situations as demonstrated by the following examples: an outbreak of hookworm infection during military operations in Grenada (1), an outbreak of malaria in US Marines serving in Somalia (2), an outbreak of Norwalk-like virus (NLV) in US ground troops deployed to Iraq in 1991 (3) and an outbreak of influenza virus in camps during World War I that killed approximately 100,000 troops (4). In addition, gastrointestinal outbreaks caused by *Norovirus*, parasites and bacteria among military officers boarding navy ships have been widely reported (5).

To date, these outbreaks have been managed and controlled in a timely manner. However, outbreaks of microbial toxins or bio-safety level (BSL) 3 and 4 pathogens, such as *Mycobacterium tuberculosis*, Ebola, *etc.* or with not-yet-characterized newly emerging pathogens of unknown risk classification, will require transportation of samples to established laboratories for identification. However, strict regulations put in place by the International Air Transport Association (IATA, www.iata.org) make the international transport of biological specimens and isolates challenging. Nevertheless, transfers to laboratories in the home country may be desirable, in particular, if new pathogens of as yet unknown pathogenic potential are detected.

Next generation sequencing (NGS) is one of the most promising diagnostic approaches, which allows for rapid sequencing and assembly of whole pathogen genomes [9, 10] and for the non-targeted detection of pathogens directly from primary sample material [11]. NGS-based ultra-deep sequencing from biological samples or cultured strains is sufficiently deep as to allow for the reconstruction of the whole pathogen genomes. In addition, upon successful sequence analysis at the deployment site, sequences are more easily transferred to the home country through the internet than as physical microbial isolates. Scientists in the receiving laboratory are able to analyze the raw sequence data and identify the pathogen under investigation.

In this review article, we discuss the importance of applying NGS and synthetic biology during military deployments.

## Review

### Next-Generation DNA sequencing

Since the heyday of Sanger sequencing, sequencing technologies have undergone considerable progress, thus currently allowing for the affordable sequencing of whole pathogen genomes [12, 13]. Although Sanger sequencing, based on slab gel electrophoresis and automatic multicapillary electrophoresis systems, is still used for the analysis of the human genome [14], new approaches allow for analysis of whole genomes within hours to few days [14, 15]. Such NGS technologies include cyclic array, hybridization-based, nanopore and single molecule sequencing [14] and have been commercialized by companies such as 454 Life Sciences, Applied Biosystems, Danaher Motion Co., Helicos Biosciences, Illumina, Oxford Nanopore Technologies and Pacific Biosciences.

The technological principles of the different NGS approaches have been detailed elsewhere. In short, cyclic-array sequencing is based on library preparation by random DNA fragmentation, ligation of common adaptor sequences, spatial gathering of clonally clustered amplicons on planar substrates or on microbeads, emulsion amplification, and enzyme catalyzed elongation followed by spectral imaging based on sequencing-by-synthesis technology [16]. So-called 454 sequencing [17] combines PCR-based cloning and pyrosequencing [18]. Bridge PCR-based sequencing is based on the preparation of clonal libraries, immobilization of forward and reverse primers onto solid planar substrates using flexible linkers and cyclic single base extension with subsequent interrogation of the results [19]. The sequencing-by-hybridization approach begins with clonal sequencing in emulsion PCR, followed by differential hybridization of labeled nucleic acid fragments to an array of oligonucleotide probes [20]. Hybridized-primer/template pairs for single molecule sequencing are produced by the preparation of large quantities of poly (dA), possessing single stranded DNA templates, with subsequent hybridization to tethered poly (dT) primers on glass substrates [21]. Last but not least, the nanopore approach allows for massively parallel, high throughput sequencing of label-free single molecules by direct electrical identification of DNA bases [22]. Ongoing automation has led to further dramatic decreases in sequencing costs from approximately 100,000 € per human genome to as low as 1000 € [14].

### Obtaining high-quality sequences by NGS – a pre-requisite for subsequent pathogen design

If sufficient sequencing depth can be guaranteed, NGS allows for a non-specific screening for infectious agents in biological samples without the need for an initial suspicion of a certain infection. In the past, associations of microbial pathogens with diseases of unknown etiology could be demonstrated [11]. Likewise, NGS technology might be used for the detection of completely unknown pathogens within samples. Particularly in times of rapidly decreasing sequencing costs, the use of laser-printer sized bench-top NGS devices at deployment sites might be a realistic goal over the intermediate term. However, sequence quality is not identical for all NGS approaches described above. Recently, other authors have compared the bench-top device 454 GS Junior (Roche, Basel, Switzerland), MiSeq (Illumina, San Diego, CA, USA) and Ion Torrent PGM (Life Technologies, Grand Island, NY, USA) [23]. The MiSeq (Illumina) system showed the best results regarding throughput per run and error rates, whereas both the 454 GS Junior (Roche) and the Ion Torrent PGM (Life Technologies) systems had limitations due to homopolymer-associated indel errors [23]. Technological development is still ongoing in this field.

Sophisticated down-stream data analysis is at present a further limitation of broad applications of NGS technology in the routine diagnostic setting. Data analysis is still relatively challenging, not user-friendly, and requires large teams of skilled scientists, bioinformaticians, and technical assistants [24]. Over the intermediate term, ongoing automation might allow NGS to become user-friendlier and thus useful for routine diagnostic situations.

### Reconstruction of pathogen genomes

Based on the transmitted sequences, circular DNAs (cDNAs) can be synthesized *de novo* by assembling oligonucleotides of plus and minus strand polarity in the home country. The underlying technology, called “DNA shuffling”, was described as early as 1995. In short, 40-base-pair oligonucleotides with overlapping ends are chemically synthesized; this method is comparable to primer synthesis for PCR. Thermal cycling and polymerase are subsequently used to link the oligonucleotides. If the resulting sequence is designed with flanked ends that match a plasmid palindrome sequence, they can be inserted into a plasmid, which is then inserted into cells for further replication [25]. If the inserted sequence codes for a virus, it can be assembled within the cell as shown for the examples described below.

Using transformation, genetic elements can be incorporated into a variety of different cells that serve as expression vectors. For example, bacteria have acquired the ability to express foreign DNA naturally since the origins of life more than three billion years ago and incorporation of transposable genetic elements into yeast cells is a well-established procedure in biotechnology [26, 27].

### Successfully revitalized pathogens

In 2002, replicative infectious polioviruses were artificially created in a cell-free *in vitro* environment based solely on synthetic poliovirus cDNA molecules, a milestone in medical science. This was the first demonstration of the synthesis of an infectious agent using *in vitro* biochemical means to follow a written sequence. Currently, complete synthetic design of a virus can be performed within 8 weeks [28, 29].

In addition to whole pathogen genomes, it is also possible to clone single genes in bacterial vectors, allowing for the design of reassortant strains using reverse genetics methods. Such cloning is particularly suitable for pathogens with segmented genomes, e.g., influenza viruses [30]. Such new designs are usually of use as vaccine candidates. Similarly, virus-like particles (VLP) containing triple hemagglutinin for influenza vaccination were derived from recombinant baculovirus [31].

As an example of the revitalization of pathogens for pathogenicity research, the 1918 influenza virus, the causative agent of the so-called “Spanish flu”, was synthesized by DNA shuffling [32, 33]. Sequence information of high pathogenicity linked to 3 loci of the viral genome was preserved in permafrost soil for more than 90 years [32]. The experiments impressively demonstrated that no virus is truly extinct as long as its genetic information is still available.

Much attention was paid to the introduction of synthetic pathogens with artificially altered pathogenicity. In 2001, a recombinant ectromelia virus expressing mouse interleukin 4 was introduced. Co-infection with IL-4-expressing ectromelia virus and mousepox virus leads to suppression of cytolytic lymphocyte responses and consecutive breakdown of resistance to mousepox in laboratory animals [34, 35]. The experiments resulted in public fear of the dual-use potential of genetically modified organisms (GMO) [36].

In contrast, modified viruses can also have beneficial effects that can be used for therapeutic strategies over the intermediate or long term, e.g., modified oncolytic herpes simplex virus in combination with interleukin-12 led to lysis of glioblastoma cells [37] and a modified adenovirus was shown to influence tumor cell growth [38].

Coxsackievirus B3 (CVB3) M2 is an example of an RNA-virus that was sequenced and translated into cDNA. For revitalization, the pCVB3-M2 plasmid can be easily transfected into HeLa cells [39]. Use of plasmid-generated viruses allows the easy construction of chimeric viruses [40], which can be used to determine replication patterns [40] and cell tropism [41].

The *de novo in vitro* synthesis of bacterial pathogens based on their sequence information has been demonstrated in other experiments [42–44]. For example, synthetic bacterial design by mega-cloning is based on amplification of circular genomes in yeast cells [45].

### Synthetic biology – a tool for the future

The underlying up-to-date options available for synthetic biology have recently been extensively reviewed [46]. These options comprise techniques of whole genome assembly, which is usually performed in yeast cells [47], and activation of artificially designed genomes [44]. *De novo* genome synthesis, which has been used to design a 583-kb-genome of *Mycoplasma genitalum* [42] and a 1.1 Mb-genome of *Mycoplasma mycoides* [48], requires a variety of synthesis and assembly steps, which have been detailed elsewhere [42, 48–53]. However, if the desired sequences exist in nature, or are available as templates, amplification of the required sequences for the assembly process is more convenient and cost-effective than *de novo* design [46].

Although *in vitro* assembly of large-sized genetic elements, e.g., by Gibson Assembly with amplification by PCR or rolling-circle amplification approaches, is possible in principle, it is prone to a higher mutation frequency than cloning in yeast cells. *In vivo* genome assembly in yeast cells has therefore been chosen for the *de novo* synthesis of the bacterial genomes described above [42, 48]. Factors that affect the cloning capacity of whole genomes in yeast cells include the absence of toxic expression, genome size and GC content [46].

After the design and assembly of a genome, its activation requires a suitable environment, which is usually provided by implantation into appropriate enucleated target cells. If the environment is not suitable, activation fails and the recipient cell is not transformed. Factors that can affect genome activation include the presence or absence of nuclease activity, the presence of a cell wall, genome size, and similarity between donor and recipient species [46]. Cell-free genome activation will be the goal of future research but has not yet been achieved for complex bacterial genomes [54–56].

Although the approaches taken by synthetic biologists ultimately target design and modification of new organisms [46], such highly ambitious goals are unnecessary for diagnostic microbiology in the military deployment setting.

### Discussion

Several scientific milestones were necessary to come to the current point of synthetic design of microbial pathogens, beginning with the understanding of bacterial and viral gene transfer [14]. Milestones for the deciphering of the principles of gene transfer include description of the transforming principle by Frederick Griffith in 1928, identification of DNA as the carrier of genetic information by Avery, MacLeod, and McCarty in 1944, identification of transduction as a mechanism of genetic transfer by Lederberg in 1952, description of the double-helix structure of DNA by Watson and Crick in 1953, identification of inherited genetic mutation as a result of viral infection in 1961 by Temin, the first documented heritable gene transfer in mammalian cell lines in 1962 by Szybalski, demonstration of virus-mediated gene transfer by Rogers & Pfuderer in 1968, the first gene transfer into humans in 1989 by Rosenberg, the first approved gene-therapy-based product for clinical use in China in 2003, and a first successful clinical phase III gene therapy trial in the European Union in 2009 [14]. Following from this research, introduction of extraneous nucleic acid sequences into cells became possible, culminating in *de novo* design of viruses based on their nucleic acid sequence [17].

Revitalization of microorganisms based on their sequence information is of importance for diagnostic purposes if the pathogenic potential of an isolated strain is to be assessed under *in vitro* conditions. In a military deployment setting, a newly isolated strain from a deployment site can be inactivated to be of no more harm for medical laboratory scientists who have to handle it under restricted deployment conditions. After sequencing and transferring the sequence data to the home country, the strain can be revitalized under BSL-3 or BSL-4 laboratory conditions using *de novo* synthesized DNA molecules. Inactivation at the site of isolation with subsequent revitalization at a site that is safe for further investigation will considerably reduce the risk of nosocomial laboratory infections on deployment.

To the authors’ best knowledge, at present, no military medical service uses technologies to inactivate a pathogen, to analyze it using NGS to synthesize cDNAs, and to revitalize it at another place under safe laboratory conditions. Such technological approaches are currently considered scientific top-end and are very expensive. Nevertheless, evaluation of such technologies for diagnostic purposes by the armed medical services seems desirable. Standardization is also widely missing. Accordingly, any future diagnostic use of technologies described herein will require considerable evaluation and standardization work and will have to account for sequencing errors [12], as detailed above, due to insufficiencies in the existing technology.

## Conclusions

A structured evaluation and standardization of diagnostic *in vitro* revitalization of inactivated pathogens by the armed medical services will require a joint multi-national approach to be cost-effective. The potentials of such an approach for international diagnostic and scientific cooperation, in the case of the appearance of a harmful new pathogen, might be worth the effort. Over the long term, microbiological strain collections could be completely replaced by sequence databases, and international strain exchanges could be realized by a simple mouse click.

## Availability of supporting data

All relevant data for the assessment are presented in the paper.

## Abbreviations

BSL: Bio-safety level
cDNA: Circular deoxyribonucleic acid
DNA: Deoxyribonucleic acid
GMO: Genetically modified organism
IATA: International Air Transport Association
NGS: Next generation sequencing
PCR: Polymerase chain reaction
VLP: Virus-like particles
